# Myristoylation profiling in human cells and zebrafish

**DOI:** 10.1016/j.dib.2015.06.010

**Published:** 2015-07-02

**Authors:** Malgorzata Broncel, Remigiusz A Serwa, Paulina Ciepla, Eberhard Krause, Margaret J. Dallman, Anthony I. Magee, Edward W. Tate

**Affiliations:** a Department of Chemistry, Imperial College London, Exhibition Road, London SW7 2AZ, UK; b Leibniz-Institut für Molekulare Pharmakologie (FMP), Robert-Roessle-Str. 10, 13125 Berlin, Germany; c Department of Life Sciences, Imperial College London, Exhibition Road, London SW7 2AZ, UK; d National Heart and Lung Institute, Imperial College London, Exhibition Road, London SW7 2AZ, UK

**Keywords:** Protein modification, Myristoylation, Tagging by substrate, Modification site

## Abstract

Human cells (HEK 293, HeLa, MCF-7) and zebrafish embryos were metabolically tagged with an alkynyl myristic acid probe, lysed with an SDS buffer and tagged proteomes ligated to multifunctional capture reagents via copper-catalyzed alkyne azide cycloaddition (CuAAC). This allowed for affinity enrichment and high-confidence identification, by delivering direct MS/MS evidence for the modification site, of 87 and 61 co-translationally myristoylated proteins in human cells and zebrafish, respectively. The data have been deposited to ProteomeXchange Consortium (Vizcaíno et al., 2014 *Nat. Biotechnol*., 32, 223–6) (PXD001863 and PXD001876) and are described in detail in Multifunctional reagents for quantitative proteome-wide analysis of protein modification in human cells and dynamic protein lipidation during vertebrate development׳ by Broncel et al., *Angew. Chem. Int. Ed*.

**Specifications table**

**Table:** 

Subject area	*Biology, chemical proteomics, post-translational modification*
More specific subject area	*Protein myristoylation*
Type of data	*Proteomics, tandem mass spectrometry*
How data was acquired	*Mass spectrometry using a Q Exactive mass spectrometer (Thermo Fisher Scientific)*
Data format	*Raw LC–MS/MS, searched with PEAKS7 and MaxQuant 1.5*
Experimental factors	*Samples were processed using tagging by substrate methodology*
Experimental features	*Tryptic peptides were detected by LC–MS/MS and identified by database searches with two independent proteomics platforms*
Data source location	*http://proteomecentral.proteomexchange.org/cgi/GetDataset?ID=PXD001863*
	*http://proteomecentral.proteomexchange.org/cgi/GetDataset?ID=PXD001876*
Data accessibility	*The data have been uploaded to the ProteomeXchange Consortium*[1]*(PXD001863 and PXD001876) and are described in detail by Broncel* et al. [2]

**Value of the data**

• The largest repository for myristoylated proteins in human cells.
• First database for myristoylated proteins in zebrafish.
• First quantitative description of myristoylation dynamics in a developing vertebrate.

## 1 Experimental design

**Fig. 1:**
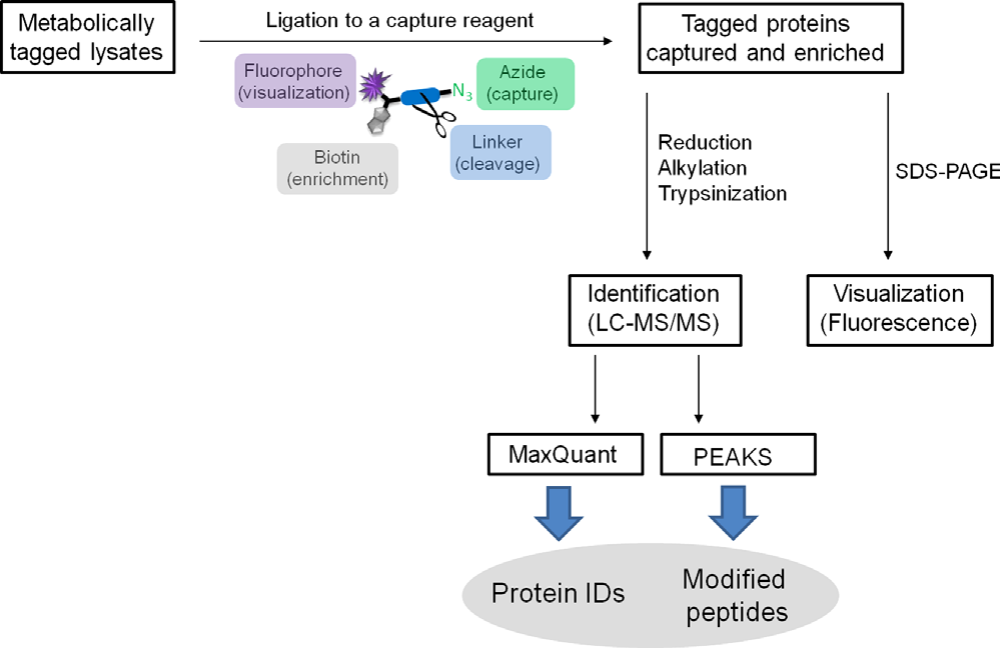
Schematic overview of the experimental design used in this study. For details of visualisation of enriched proteomes see [2].

Cells and zebrafish were metabolically tagged with an alkynyl myristic acid analogue or myristic acid control. Following lysis the tagged proteins were ligated via CuAAC to multifunctional capture reagents, [2] affinity enriched and digested to peptides before LC–MS/MS analysis (Fig. 1). Generated spectra were processed with MaxQuant [3] and PEAKS [4] software for protein identifications and lipid modified peptide discovery, respectively. Myristoylated proteins and peptides detected in this study have been deposited as PXD00186 (human) and PXD001876 (zebrafish). Quantitative data (triplex dimethyl labelling) [5] revealing myristoylation dynamics during zebrafish development was included as a part of repository submission PXD001876.

## 2 Materials and methods

### 2.1 Cell and zebrafish culture

Cells (HEK 293, HeLa, MCF-7) were maintained in DMEM+10% FBS, at 10% CO_2_, and at 37 °C. Zebrafish were maintained according to standard practices. All procedures were conformed to U.K. Home Office regulations (ASPA 1986) under Animal Project Licence no. PPL 70/7472. Adult zebrafish strains AB were kept at 28 °C on 14 h light and 10 h dark cycle. Embryos were obtained from natural spawnings and were maintained in system water (combination of tap and reverse osmosis water).

### 2.2 Metabolic tagging and lysis

Cells: the medium was supplemented with 20 µM YnMyr or Myr for 24 h, then washed twice with Phosphate 10 mM Buffered pH 7.4 Saline 0.154 M (PBS) and lysed on ice using the following lysis buffer: PBS, 0.1% SDS, 1% Triton X-100, 1× EDTA-free complete protease inhibitor. Lysates were kept on ice for 20 min, and then centrifuged at 17,000*g* for 20 min and supernatants collected and stored at −80 °C. The Bio-Rad DC Protein Assay was applied to determine protein concentration.

Zebrafish: embryos were placed in system water containing 0.0001% methylene blue (zebrafish water). The embryos were treated as described in Laughlin et al. [6] with minor adjustments. At 4–5 h post-fertilization (hpf), 48 hpf and 96 hpf the embryos were dechorionated with pronase (1 mg/mL, 7 min), and washed with zebrafish water. The cleaning process was repeated four times to remove most of the chorions. The embryos were transferred into 1% agarose-coated wells of a 48-well plate containing 250 μL of zebrafish water supplemented with 20 µM YnMyr or 20 µM Myr and incubated for 24 h before they were euthanized with MS-222 solution (250 mg/L). The embryos were transferred to 1.5 ml Eppendorf tubes and deyolked using a protocol described in Link et al. [7] and further treated as described in Hinz et al. [8] with adjustments. The embryos were washed with 200 μL ice-cold lysis buffer: PBS, 2× EDTA-free complete protease inhibitor. The solution was removed and replaced by fresh lysis buffer (100 μL). Zebrafish embryos were homogenised with a Kimble^®^ Kontes Disposable pellet pestle with cordless motor. SDS (1%) and Benzonase^®^ Nuclease (0.5%) were added and the lysates were vortex-mixed at RT for 5 min and heated at 95 °C for 10 min. Lysates were allowed to cool down on ice for 5 min, diluted with lysis buffer to 250 μL and supplemented with Triton X-100 (0.2%). Lysates were centrifuged (16,000*g*) at 4 °C for 10 min, supernatants collected and used for further experiments or stored at −80 °C.

### 2.3 CuAAC and sample preparation for MS-based proteomics

Cells: 0.4 mg (small scale) or 2 mg (large scale) of proteins at the concentration of 2 mg/mL was incubated with a premixed solution of a capture reagent (0.1 mM), CuSO_4_ (1 mM), TCEP (1 mM) and TBTA (0.1 mM). The samples were vortex-mixed at RT for 1 h before the addition of EDTA (10 mM), methanol (4 v/v), chloroform (1 v/v), and water (3 v/v). The samples were vortex-mixed briefly, centrifuged (10,000*g*) at RT for 20 min to produce protein pellets, which were reconstituted in 1% SDS in PBS at 2 mg/mL, and the precipitation step was repeated. Following centrifugation, the pellets were washed with methanol and briefly dried on air. The pellets were reconstituted in 2% SDS in PBS at 5 mg/mL, and PBS was added to dilute the samples to 1 mg/mL. Bioorthogonally labelled proteins were enriched on prewashed NeutrAvidin agarose resin (50 µL slurry/1 mg of lysate) for 2 h at RT. Following the removal of supernatants the beads were washed with 1% SDS in PBS (3×), 4 M urea in 50 mM AMBIC (2×) and 50 mM AMBIC (5×). The beads were treated with 5 mM DTT for 30 min at 55 °C, and then with 10 mM iodoacetamide for 30 min at RT, in the dark. The beads were washed with 50 mM AMBIC twice after each step. Protein digestion was initiated upon addition of trypsin or chymotrypsin, 1/1000 w/w protease to pre-pull-down protein, and samples were incubated overnight at 37 °C or 25 °C, respectively. The samples were briefly centrifuged, diluted 1:1 with 0.1% TFA, and stage-tipped according to a published protocol. [9] Peptides were eluted from the sorbent (SDB-XC, from 3 M) with 70% acetonitrile in water, followed by SpeedVac-assisted solvent removal, reconstitution in 0.5% TFA, 2% acetonitrile in water, and transfer into LC–MS sample vials. All samples were prepared in triplicates.

Zebrafish: samples were processed as described for cell-based experiments with adjustments. For label free experiments, CuAAC reaction was carried out with 1.5 mg of proteins (mix of 3×0.5 mg^2^ from 24 h pulse tagging experiments ending at 24, 72 and 120 hpf). For dimethyl labelling experiments, CuAAC reaction was carried out with 0.2 mg of proteins. Triplex dimethyl labelling was performed according to a published protocol, [5] and the samples (24 h pulse tagging ending at 24, 72 and 120 hpf) were mixed 1:1:1 prior to the SpeedVac-assisted solvent removal. All experiments involved biological triplicates.

### 2.4 LC–MS/MS analysis

The analysis was performed using an Acclaim PepMap RSLC column 50 cm×75 μm inner diameter (Thermo Fisher Scientific) using a 2 h acetonitrile gradient in 0.1% aqueous formic acid at a flow rate of 250 nL/min. Easy nLC-1000 was coupled to a Q Exactive mass spectrometer via an easy-spray source (all Thermo Fisher Scientific). The Q Exactive was operated in data-dependent mode with survey scans acquired at a resolution of 75,000 at *m/z* 200 (transient time 256 ms). Up to 10 of the most abundant isotope patterns with charge +2 or higher from the survey scan were selected with an isolation window of 3.0 *m/z* and fragmented by HCD with normalised collision energies of 25. The maximum ion injection times for the survey scan and the MS/MS scans (acquired with a resolution of 17,500 at *m/z* 200) were 20 and 120 ms, respectively. The ion target value for MS was set to 10^6^ and for MS/MS to 10^5^, and the intensity threshold was set to 8.3×10^2^.

### 2.5 Proteomics data analysis with MaxQuant

The data were processed with MaxQuant [3] (version 1.5.0.25) and the peptides were identified from the MS/MS spectra searched against human complete proteome (Uniprot, September 2014) [10] using Andromeda [11] search engine. Cysteine carbamidomethylation was selected as a fixed modification and methionine oxidation as a variable modification, the minimum peptide length was set to 7 amino acids. Myristoylated peptide search was performed applying the following variable peptide N-terminal modifications that corresponded to the added masses of alkynyl myristic acid ligated to multifunctional capture reagents: +463.2907; +633.4326; +406.2692; +520.3373; +624.3999, [2] the minimum peptide length was reduced to 6 amino acids. For *in silico* digests of the reference proteome the following peptide bond cleavages were allowed: arginine or lysine followed by any amino acid (trypsin); phenylalanine, tyrosine or tryptophan followed by any amino acid (chymotrypsin). Up to two missed cleavages were allowed. The false discovery rate was set to 0.01 for peptides, proteins and sites. Other parameters were used as pre-set in the software. “Unique and razor peptides” mode was selected to allow identification and quantification of proteins in groups. Label-free quantification (LFQ) was performed using a built-in algorithm enabling the ‘Match between runs’ option (time window 0.7 min). The experiment comprised three replicates treated with YnMyr (and three replicates treated with Myr, where applicable). Triplex dimethyl labelling experiments in MaxQuant were performed using the built-in quantification algorithm enabling the ‘Match between runs’ (time window 0.7 min) and ‘Re-quantify’ features. Light (+0 Da), medium (+4 Da) and heavy (+8 Da) intensities were selected for a triplex experiment.

### 2.6 Proteomics data analysis with PEAKS suite

The data for modified peptide discovery were processed with PEAKS7, [4] which as a default performs de novo peptide sequencing prior to database searches, in order to improve the accuracy of the results. The software also searches for common PTMs (PEAKS PTM) and point mutations (SPIDER). Samples originating from cell lines and zebrafish experiments were searched against the same reference Uniprot [10] Homo sapiens and *Danio rerio* databases that were used in MaxQuant analyses. Trypsin (specific, up to three missed cleavages allowed) was selected for database searches, and no enzyme was chosen in de novo searches (up to 5 candidates per spectrum reported). The maximal mass error was set to 5 ppm for precursor ions and 0.01 Da for product ions. Carbamidomethylation was selected as a fixed modification, and methionine oxidation as well as the lipid-derived adduct (+463.2907 Da) to any amino acid at peptide N-terminus were set as variable modifications. The maximal number of modifications per peptide was set as five. The false discovery rate was set to 0.01 for peptides and minimum of 1 unique peptide per protein was required. For N-terminally myristoylated peptides, b1 ions were required.
